# Phenotypic and Genotypic Properties of Fluoroquinolone-Resistant, *qnr*-Carrying *Escherichia coli* Isolated from the German Food Chain in 2017

**DOI:** 10.3390/microorganisms9061308

**Published:** 2021-06-16

**Authors:** Katharina Juraschek, Carlus Deneke, Silvia Schmoger, Mirjam Grobbel, Burkhard Malorny, Annemarie Käsbohrer, Stefan Schwarz, Diana Meemken, Jens Andre Hammerl

**Affiliations:** 1 German Federal Institute for Risk Assessment (BfR), Max-Dohrn Str. 8-10, 10589 Berlin, Germany; Carlus.Deneke@bfr.bund.de (C.D.); silvia.schmoger@bfr.bund.de (S.S.); mirjam.grobbel@bfr.bund.de (M.G.); Burkhard.Malorny@bfr.bund.de (B.M.); annemarie.kaesbohrer@bfr.bund.de (A.K.); 2 Unit for Veterinary Public Health and Epidemiology, University of Veterinary Medicine, Veterinaerplatz 1, 1210 Vienna, Austria; 3 Department of Veterinary Medicine, Institute of Microbiology and Epizootics, Freie Universität Berlin, 14163 Berlin, Germany; Stefan.Schwarz@fu-berlin.de; 4 Department of Veterinary Medicine, Institute of Food Safety and Food Hygiene, Freie Universität Berlin, 14163 Berlin, Germany; diana.meemken@fu-berlin.de

**Keywords:** *E. coli*, typing, genomes, plasmid, livestock, food, fluoroquinolones

## Abstract

Fluoroquinolones are the highest priority, critically important antimicrobial agents. Resistance development can occur via different mechanisms, with plasmid-mediated quinolone resistance (PMQR) being prevalent in the livestock and food area. Especially, *qnr* genes, commonly located on mobile genetic elements, are major drivers for the spread of resistance determinants against fluoroquinolones. We investigated the prevalence and characteristics of *qnr*-positive *Escherichia (E.) coli* obtained from different monitoring programs in Germany in 2017. Furthermore, we aimed to evaluate commonalities of *qnr*-carrying plasmids in *E. coli*. We found *qnr* to be broadly spread over different livestock and food matrices, and to be present in various sequence types. The *qnr*-positive isolates were predominantly detected within selectively isolated ESBL (extended spectrum beta-lactamase)-producing *E. coli*, leading to a frequent association with other resistance genes, especially cephalosporin determinants. Furthermore, we found that *qnr* correlates with the presence of genes involved in resistance development against quaternary ammonium compounds (*qac*). The detection of additional point mutations in many isolates within the chromosomal QRDR region led to even higher MIC values against fluoroquinolones for the investigated *E. coli*. All of these attributes should be carefully taken into account in the risk assessment of *qnr*-carrying *E. coli* from livestock and food.

## 1. Introduction

Antimicrobial resistance (AMR), especially against the highest priority, critically important substances (e.g., quinolones and fluoroquinolones) is a global threat for humans and animals. Food-producing animals are considered an important reservoir of AMR-carrying bacteria [1,2]. Therefore, annual monitoring programs in the EU are conducted to observe trends in the development and dynamics of resistances in specific target animals. Commensal *Escherichia* (*E.*) *coli* serves as an indicator bacterium among *Enterobacteriaceae* for estimating changes in the prevalence of resistance genes in food and livestock in European countries.

Quinolones and fluoroquinolones, further named (fluoro)quinolones, are antimicrobial agents, considered as clinically highly important substances [3], and are used for the treatment of animal infections and human diseases in Europe. In the last years, EFSA notified a steadily increasing trend in (fluoro)quinolone-resistant bacteria, isolated from food-producing animals. Therewith, a high proportion of *Salmonella enterica* and *E. coli*, mainly isolated from poultry, were classified as not susceptible against ciprofloxacin. Next to the livestock sector, the human sector also registered an increase in ciprofloxacin resistance from 1.7% (in 2016) to 4.6% (in 2018) in certain *Enterobacteriaceae* [4]. The prevailing trend in European countries indicates a spread of (fluoro)quinolone-resistant bacteria, which poses a threat to animal and human health.

Resistance development against (fluoro)quinolones can occur via various mechanisms ranging from alterations of chromosomal genes to the acquisition of specific transferable genes. Mutations in the chromosomal elements encoding the target enzyme DNA gyrase (*gyrA*, *gyrB*) and topoisomerase IV (*parC*, *parE*) can alter the susceptibility of the isolates considerably. Other resistance mechanisms are involved in an overexpression of quinolone efflux pumps, alteration of the membrane permeability, or enzymatic inactivation of specific (fluoro)quinolones. These mechanisms can be induced by plasmid-mediated quinolone resistances (PMQR) including pentapeptide-encoding *qnr* genes, efflux pump-encoding genes (e.g., *qepA*), and the aminoglycoside acetyltransferase-coding *aac-(6′)-Ib*-*cr* gene [5]. PMQRs represent an inevitable threat, as they play an important role in the dissemination of (fluoro)quinolone resistance genes through horizontal gene transfer and the development of new (fluoro)quinolone-resistant bacteria. Hence, they are assumed accountable for the increased resistance to (fluoro)quinolones [6,7]. Horizontal gene transfer (HGT) is an efficient mechanism for adaptation of bacteria to prevailing environmental conditions. The exchange of resistance genes is a common response of bacteria to overcome antimicrobial selection pressures. Among them, *qnr* represents a highly prevalent PMQR gene in livestock with a broad overall distribution [8]. It has frequently been reported that *qnr* genes in *Enterobacteriaceae* increase (fluoro)quinolone resistance by enhancing the degree of resistance at which they can be selected [9]. Furthermore, some reports have linked the presence of PMQRs with a successive development of chromosomal alterations in the genes *gyrA*, *gyrB,* or *parC*, known to be associated with increased (fluoro)quinolone resistance when mutated at specific positions [10,11]. In addition, *qnr* genes were often observed in combination with other mobile determinants involved in resistance development against other critically important antimicrobials (i.e., extended spectrum β-lactamases (ESBLs), carbapenemases, and colistin) [12,13,14]. The co-occurrence of genes associated with resistances against antimicrobial agents routinely used in human medicine is of great concern, as it limits the therapeutic options for treatment of infections [15]. To estimate the specific impact of *qnr*-carrying isolates for the emergence and dissemination of (fluoro)quinolone-resistant *E. coli* in livestock and food, a deeper understanding of the occurrence, genetic variability, and elements involved in their spread is needed.

This study was conducted to assess the prevalence and diversity of *qnr*-carrying isolates among (fluoro)quinolone-resistant, commensal *E. coli* gained during the annual German AMR monitoring in livestock and food in 2017. These isolates were characterised in detail for their resistance phenotype and genetic characteristics. Furthermore, the commonality of plasmids carrying *qnr* genes along with their diversity and transmissibility were determined. Finally, a potential association of *qnr*-genes with (fluoro)quinolone resistance enhancing point mutations among German livestock *E. coli* was evaluated.

## 2. Materials and Methods

### 2.1. Bacterial Isolates and Culture Conditions

In total, 2799 *E. coli* from different food and livestock sources [16], especially from faecal samples of deer and fattening pigs, from cecum contents of fattening pigs and veal calves, as well as samples from pork, veal, and game meat where analysed. Samples from faecal and cecum sources are analysed as the same source in this study. All isolates were recovered during the German AMR monitoring of commensal (ZoMo, unselective cultivation conditions) and ESBL and/or AmpC-producing *E. coli* from food and livestock (ESBL-monitoring, selective cultivation conditions using cefotaxime) in 2017. The isolates were investigated according to the European Commission Implementing Decision 2013/652/EU in the National Reference Laboratory for Antimicrobial Resistance (NRL-AR). The isolates constitute the positive findings of a representative collection of samples taken in all 16 German federal states. If not stated otherwise, all isolates were cultivated in lysogeny-broth-based media for 16–18 h at 37 °C for further characterisation.

### 2.2. Antimicrobial Susceptibility Testing

Minimum inhibitory concentrations (MIC) were determined by using broth microdilution according to EUCAST recommendations on a standardised European antimicrobial test panel (EUVSEC/EUVSEC2; Sensititre™, TREK Diagnostic Systems, Altrincham, Cheshire, UK). The tested antimicrobials covered the substances and ranges fixed in the European Commission Implementing Decision No. 2013/652/EU [17]. The following antimicrobial agents were used in ranges as specified: ampicillin (1 to 64 mg/L), azithromycin (2 to 64 mg/L), cefepime (0.06 to 32 mg/L), ciprofloxacin (0.015 to 8 mg/L), colistin (1 to 16 mg/L), ertapenem (0.015 to 2 mg/L), cefoxitin (0.5 to 64 mg/L), gentamicin (0.5 to 32 mg/L), imipenem (0.12 to 16 mg/L), meropenem (0.03 to 16 mg/L), nalidixic acid (4 to 128 mg/L), cefotaxime (0.25 to 64 mg/L), ceftazidime (0.25 to 128 mg/L), temocillin (2 to 128 mg/L), tetracycline (2 to 64 mg/L), tigecycline (0.25 to 8 mg/L), trimethoprim (0.25 to 32 mg/L), chloramphenicol (8 to 128 mg/L), sulfamethoxazole (8 to 1024 mg/L), cefotaxime/clavulanic acid (0.06/4 to 64/4 mg/L), and ceftazidime/clavulanic acid (0.12/4 to 128/4 mg/L). For quality assessment, the *E. coli* strain ATCC 25,922 was included. MIC values were interpreted according to EUCAST epidemiological cut-off values (ECOFFs) [18].

### 2.3. Molecular Screening on qnr Genes

Genomic DNA from isolates exhibiting a non-wild-type phenotype for nalidixic acid (NAL ≥ 16 mg/L) and/or ciprofloxacin (CIP ≥ 0.06 mg/L) were subjected to boiling DNA preparation [19]. The DNA extracts were used for molecular screening of *qnr* genes. PCR amplification for detecting *qnrA*, *qnrB*, *qnrC*, *qnrD*, *qnrS*, and *qnrVC* was conducted using primers and conditions as previously described [20,21] (Table S1). Product amplification was performed in a Bio-Rad CFX96 Touch Real-Time PCR Detection System (Bio-Rad, Feldkirchen, Germany).

### 2.4. Determination of Isolate-Specific Macrorestriction Patterns and Plasmid Profiles

For determination of the genetic relationship between isolates, macrorestriction profiles using pulsed-field gel electrophoresis (PFGE) according to the PulseNet laboratory protocol [22] were performed. For digestion, the restriction endonuclease XbaI (10 U/µL, Thermo Fischer Scientific, Darmstadt, Germany) was used. For plasmid profiling, bacteria were treated with S1 nuclease (180 U/µL, Thermo Fischer Scientific) and S1-PFGE was conducted as previously described [22]. Separation of DNA was conducted on a CHEF-DR III system (Bio-Rad Laboratories, Madrid, Spain). The *Salmonella enterica* (H9812) serovar Braenderup was used as a molecular weight standard for size determination. Detection of *qnr* gene-carrying fragments was performed by Southern blotting and DNA-DNA hybridisation of S1-PFGE agarose gels. Hybridisation was conducted using digoxigenin-labelled (Roche Diagnostics, Mannheim-Penzberg, Germany) PCR probes of *qnr* genes and were prepared as previously described [23] (Table S1).

### 2.5. Whole-Genome Sequencing (WGS) and Bioinformatics Analysis

Genomic DNA of the isolates was prepared using the PureLink Genomic DNA Mini Kit (Invitrogen-Thermo Fisher, Schwerte, Germany) according to the manufacturer’s recommendation. The sequencing library was generated with the Nextera DNA Flex Library Preparation Kit (Illumina^®^, San Diego, CA, USA) as previously described [24]. Short-read, paired-end, whole-genome sequencing was performed in 2 × 151 cycles with the Illumina^®^ NextSeq™ 500/550 Mid Output Kit v2.5 (300 Cycles). After trimming of reads with aquamis (version 1.33) [25], unicycler (version 0.4.4) [26] was used for de novo assembly of raw reads. Quality assessment of genome assemblies was conducted using QUAST 5.0.2 [27]. Assembled contigs were analysed for virulence factors and resistance genes as well as for plasmid markers (i.e., replicon types) with AMRfinder (version 3.6.7) and its database [28] and abricate (version 0.9.8) [29] each, through bakcharak [30]. Cluster analyses and sequence alignments were conducted using PATRIC with the RASTtk-enabled Genome Annotation Service and default parameters [31] for the service “Codon Tree”. Visualisation was conducted in R with the packages ggplot2 (version 3.3.0) [32], ggtree (version 1.4.11), and treeio (version 3.10) [33].

The PointFinder tool [34] and the deposited database (updated—2 July 2019; access date—25 June 2020) were used to identify alterations in chromosomal genes that were confirmed to be associated with (fluoro)quinolone resistances for *E. coli*. In-silico-based multilocus sequence typing (MLST, according to the Achtmann scheme) was conducted using bakcharak [30] and the pubMLST database [35]. The screening for respective Inc groups was based on the PlasmidFinder database [36]. The detection of resistance genes was conducted with ResFinder 4.1 [37].

To determine the diversity of *qnr*-carrying plasmids, a reference database comprising all accessible *qnr* plasmid genomes of the NCBI RefSeq database (access date—17 April 2020) was developed. All available plasmids were checked for completeness through the keywords “complete sequence” or “complete plasmid”. Abricate was performed with the NCBI AMRfinder database to screen for *qnr* plasmids. The resulted database was used for the subsequent reference search with RefSNPer [38] as described elsewhere [39]. The trimmed reads of all individual isolates were mapped to each reference plasmid (using bowtie2 [40], version 2.3.5). Subsequently, the coverage breadth and depth of each reference plasmid as well as the number of single-nucleotide polymorphisms (SNPs) are computed with SAMtools version 1.10 and BEDTools version 2.29.0 [41], thus providing the *qnr* plasmids that most closely match each isolate.

### 2.6. Analysis and Statistics

Data analysis and visualisation was conducted using R (version 3.6.3). Choropleth figure was visualised with R (version 3.6.3) using the packages maptools (version 0.9-9), sp (version 1.4-0), rgeos (version 0.5-2), and rgdal (v1.4-8). The SpatialPolygonDataFrame of Germany was provided by the Bundesamt für Kartographie und Geodäsie database (https://gdz.bkg.bund.de/index.php/default/open-data/gebietseinheiten-1-2-500-000-ge2500.html, access date—24 March 2020). Dependencies between the presence of antimicrobial resistance genes were calculated in R (version 3.6.3). The occurrence of resistance genes was translated into binary data. Correlation between certain resistance determinants was predicted by Fisher’s exact test. A *ρ*-value of <0.05 was considered as a statistically significant correlation.

## 3. Results and Discussion

### 3.1. qnrS Is the Most Prevalent qnr Gene in E. coli from Livestock and Food

Antimicrobial resistance-testing (AST) revealed that 391 out of 2799 investigated isolates (14%) exhibited a non-wild-type phenotype (phenotypical resistance) against ciprofloxacin (CIP: MIC ≥ 0.06 mg/L) and/or nalidixic acid (NAL: MIC ≥ 16 mg/L) (Table S1). Of those, 80 isolates were recovered from the ESBL-monitoring and 23 from ZoMo. PCR screening revealed seven different *qnr* genes within the 103 *qnr*-positive *E. coli* (Table 1). Among them, *qnrS* (*n* = 95) was the most prevalent gene, followed by *qnrB* (*n* = 6), while *qnrA* and *qnrVC* occurred only once each. No *qnrC*- or *qnrD*-carrying isolates were detected. The rather low prevalence of (fluoro)quinolone-resistant *E. coli* of 14% in the investigated nonpoultry matrices is in good agreement with the data summarised by EFSA [42]. While CIP and NAL resistance were reported by several European countries at high levels in broiler and turkey, the EU medians in pigs and calves were rather low (6.2% and 4.2% for NAL and 7.4% and 8.4% for CIP per matrix, respectively) [43]. Regarding the detection of specific *qnr* genes, our result is in good agreement with previous reports in which *qnrS* and *qnrB* were the most frequently detected *qnr* genes in livestock sources [44,45]. In contrast to livestock, *qnrA* genes are often present in isolates from hospitalised patients [46].

**Figure 1:**
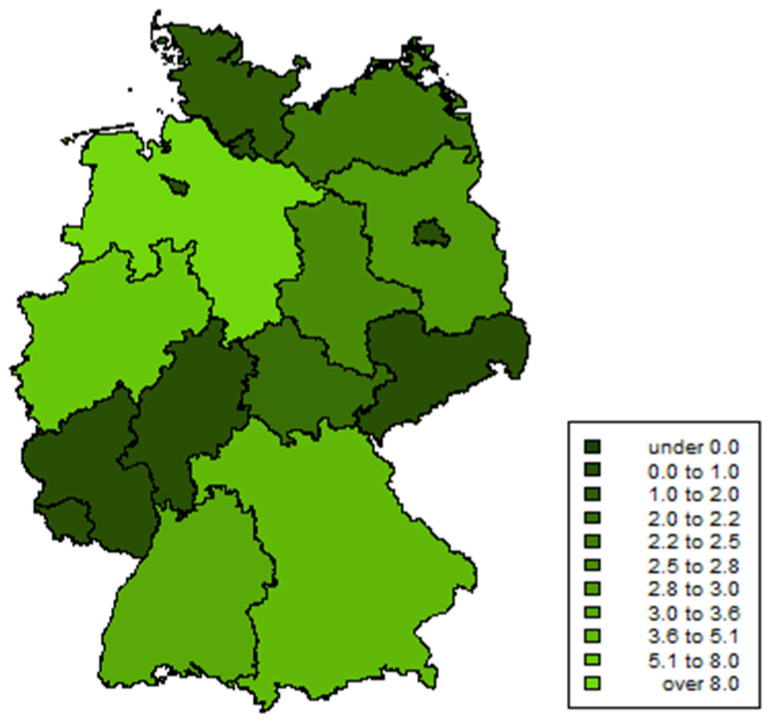
Choropleth of the proportion of (fluoro)quinolone-resistant and *qnr*-positive *E. coli* recovered during the ZoMo- and ESBL-monitoring in Germany in 2017. Prevalence was calculated as the proportion of (fluoro)quinolone-resistant and *qnr*-positive *E. coli* isolates divided by all investigated samples per federal state.

We detected *qnr*-positive *E. coli* in veal faeces (*n* = 56, 8.2% of all veal faeces samples from the ZoMo- and ESBL-monitoring in 2017), fattening pig faeces (*n* = 38, 3.6%), minced meat (*n* = 3, 4.7%), beef (*n* = 2, 5.1%), and pork (*n* = 2, 5.9%), as well as in deer faeces (*n* = 1, 0.2%) and deer meat (*n* = 1, 0.5%) (detailed data for each isolate are presented in Table S2). Overall, the highest relative proportion of (fluoro)quinolone-resistant and *qnr*-positive isolates during the monitoring program in 2017 was determined in Lower Saxony (9.3%), followed by North Rhine-Westphalia (7.9%). However, these two federal states have the highest livestock population and contributed most to the overall sample size. The prevalence for other federal states was below 5%. An overview on the regional prevalence of (fluoro)quinolone-resistant and *qnr*-positive isolates is given in Figure 1.

The analysis of the XbaI-macrorestriction patterns revealed a high phylogenetic heterogeneity of *E. coli* carrying *qnr* genes. For further typing purposes, the isolates were subjected to WGS and bioinformatics analysis. Here, 45 different sequence types (STs) were determined by in silico analysis. ST10 (21%), ST2325 (6%), and ST58 (5%) represented the predominant types. Overall, a broad distribution of *qnr*-carrying isolates in different ST-types was observed. Of ST10, 12 isolates were gained from veal faeces, nine from pork faeces, and one from veal meat. Four isolates from veal faeces, one from fattening pig faeces, and one from veal meat were assigned to ST2325. ST58 was evenly distributed between isolates from faecal samples from veal and fattening pigs. Two isolates could not be assigned to previously described STs. The observed results respond well to prevailing reports, in which *qnr* genes were found to be prevalent in ST10 isolates of livestock and of human origin and support the hypothesis on their impact as possible distributors of plasmid-associated *qnr* genes between livestock and human [47]. To the best of our knowledge, ST2325 isolates have yet not been described to be associated with *qnr* genes.

**Figure 2:**
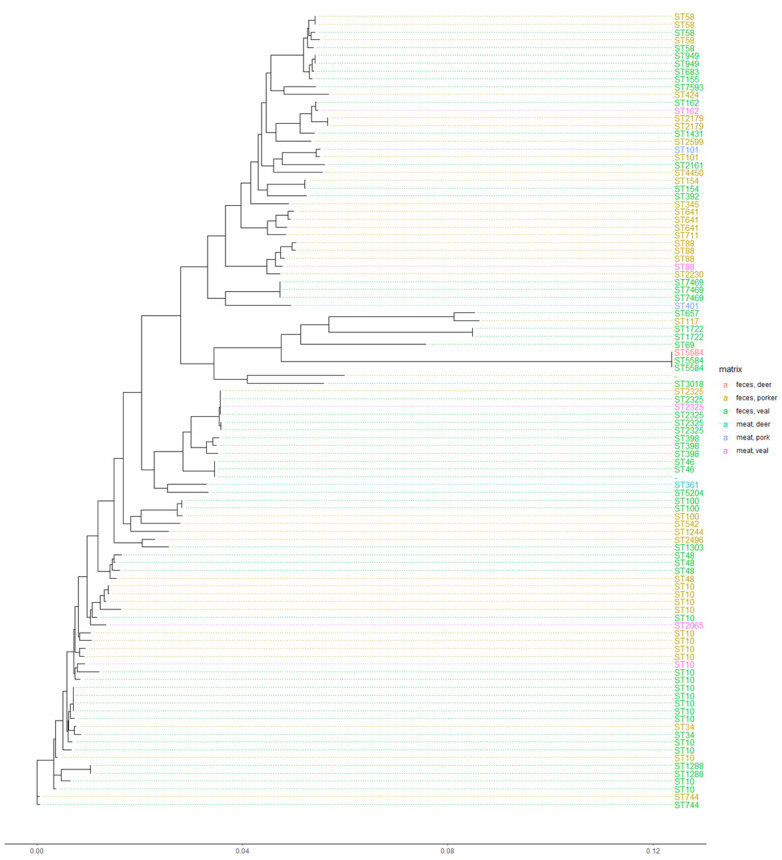
Phylogenetic relationship of (fluoro)quinolone-resistant and *qnr*-positive *E. coli* with metadata based on codon and protein differences, presented in a maximum-likelihood tree. The respective sequence type (ST) of the *E. coli* is shown as geom_tiplab and connected with dotted lines. The ST as well as the dots are coloured according to the matrix code of the recovered *E. coli*.

In Figure 2, the phylogenetic relationship of the isolates, based on WGS data of the individual isolates, is shown. As expected, the clusters correspond with the prevailing ST, but seemed not to be associated with a certain food/livestock matrix. In addition, diverse resistance profiles were observed in different clusters and STs, as well as over different matrices for the tested strains (Figure 2). Overall, we found a high diversity of *E. coli* carrying *qnr*. This widespread occurrence of *qnr* has been reported before [48]. Especially, *E. coli* of the clonal group ST10 are often associated with AMR plasmids [49] and often reported to carry *qnr*-positive plasmids [50]. Further, ST10 is characteristic for *E. coli* defined as ESBL [51]. As we analysed 80 *E. coli* isolates from the ESBL-monitoring, the observation of ST10 being related to ESBL *E. coli* was confirmed. Our findings support the current knowledge that resistance genes such as *qnr* can spread over different sources and are not restricted to certain *E. coli* sequence types.

### 3.2. qnr-Carrying E. coli Isolates Exhibit Diverse Resistance Phenotypes Including Multidrug Resistances

In antimicrobial sensitivity testing, the *qnr*-carrying *E. coli* showed highly diverse resistance profiles. Most of them exhibited a phenotypic resistance for antimicrobial classes (Table S2) other than (fluoro)quinolones, which is linked to the fact that most isolates originated from ESBL-monitoring. Considering only the 23 ZoMo-isolates, the resistant phenotype ranged from resistance to only one to five different classes. When only ESBL-monitoring isolates were analysed, the numbers ranged from three to eight different classes.

Thus, for the ESBL-monitoring isolates, 90% exhibited resistance phenotype against more than three antimicrobial classes, 79% showed resistance phenotypes against five antimicrobial agents, and 34% exhibited increased MIC values against six to eight antimicrobial agents.

In general, besides ciprofloxacin (100% from ESBL-monitoring, 91% from ZoMo), resistant phenotypes for ampicillin (99% and 91%), cephalosporine (100% and 20%), and tetracycline (80% and 52%) were most common among the investigated *E. coli*. Table 2 shows the distribution of resistant phenotypes relative to the respective matrix. This distribution is presented graphically in the Supplementary material FS1. Overall, we only detected two isolates from the ZoMo, which were only resistant to (fluoro)quinolones. Thus, our data, in which (fluoro)quinolone resistance is frequently associated with multidrug resistance in *E. coli*, coincides well with the results of other studies [52]. As a result of a direct selection pressure, the treatment of livestock with a specific antimicrobial agent supports the maintenance of resistance genes directed against this antimicrobial agent [53]. As resistance genes can occur within a multidrug-resistant isolate or on a multiresistance plasmid, disseminating through selective forces, this could enhance the prevalence of other resistance genes on the same plasmid or within the same isolate. Our result supports that there is a potential risk of coselection, maintenance, transmission, and propagation of multidrug-resistant *E. coli* and their plasmids [15]. From our findings, one could assume that multidrug-resistant clones with (fluoro)quinolone resistance exist, especially in combination with ESBL genes, which might be of special concern.

### 3.3. qnr-Carrying E. coli Isolates Are Associated with Highly Diverse Resistomes

Overall, the most abundant resistance genes of *qnr*-carrying isolates were *bla*_EC_ (accession number: A0A244BQ89) (100% of all *qnr*-carrying strains), as well as different variants of the *tet* (96%) and *bla*_CTX-M_ (74%) genes. In general, we found *qnrS1* to be in frequent connection with *tet*(34) and *tet*(A). By analysing the nucleotide-sequence of the *tet*(34) gene of *E. coli*, a sequence coverage of only 76% to the reference (accession number: A7J11_00001) was detected. Besides this defective gene, tetracycline-resistant isolates usually carried other determinants like *tet*(A) or *tet*(B). Interestingly, none of the *qnr-*harbouring *E. coli* carried a PMQR (*aac(6′)-Ib-cr*, *qepA*, or *oqxAB*) other than *qnr*.

We further split the isolates by the ZoMo- and ESBL-monitoring source. For the ZoMo isolates, we found a significant correlation for the co-occurrence of *qnrVC* with *bla*_OXA10_, *cmlA5*, and the pesticide-resistance encoding gene *qacF*. For the other *qnr* gene variants, no significant correlation for co-occurrence was detected (Table S3). No statistically significant correlation was observed for *qnrS1* and *bla*_TEM-1_ (*p*-value 0.059). However, the low number of ZoMo isolates hinders a thorough analysis for this potential correlation. When we analysed the ESBL-monitoring isolates, some co-occurrence of *qnr* and other resistance genes was detected (Table 3). Due to the selective isolation procedure, a high correlation of *qnr* and *bla* genes was observed. However, *qnrA* was rather associated with *bla*_ACC-1_ and *bla*_VIM-1_ as well as *qnrB* with *bla*_OXA-1_, while *qnrS* correlated with *bla*_CTX-M-65_ and *bla*_OXA-1_. Other co-occurring resistance genes with *qnr* are presented in Table 3. Especially, *qnrS1* was often detected in combination with multiple resistance genes as well as with the pesticide resistance encoding gene *qacE*Δ*1*.

This coexistence of multiple resistance genes can pose a higher risk, as their presence may contribute to a better adaption to different environmental conditions and enhance the persistence of the plasmid. It has been reported in previous publications that *E. coli* isolates from different livestock matrices carried both ESBL and PMQR genes, as they often coexist on the same plasmid. Mainly, a coexistence of *qnr* and *bla*_CTX-M-15_ as well as *bla*_SHV_ was previously described [12]. In general, ESBL-producing *E. coli* are an emerging public-health threat and their rise will further reduce the available treatment options in human medicine. The co-occurrence of *qnr* and ESBL genes represent another risk as the bacteria exhibit resistances against antimicrobials of two important classes. Especially, the spread of plasmids bearing resistance determinants of both antimicrobial classes will further force the development of multidrug-resistant isolates. A correlation of *qnr*-positive ESBL *E. coli* was previously reported for human sources. The presence of genes conferring resistances against two critically important antimicrobial agents on the same plasmid or within one isolate can constitute an important issue for treatment failures when using the respective antimicrobial agents for therapeutic application in hospitalised patients. As Salah et al. mentioned, these plasmid-mediated resistances highly facilitate the spread and increase their frequency [54]. They also found that every *qnr*-positive strain investigated in their study was ESBL-producing. However, as we mainly screened ESBL-preselected *E. coli*, the observation of *qnr* in these ESBL-producers presumably relates on a conditional probability. Although the coexistence of multiple PMQR genes has been described as a frequent event [52], we did not detect PMQRs other than *qnr*.

As mentioned, a significant co-occurrence of *qnrS1* and *qacE*Δ1 as well as of *qnrVC* and *qacF* was observed. These determinants confer resistance to quaternary ammonium compound disinfectants [55]. The awareness for this plasmid-associated antiseptic resistance gene is broadly present, as it enhances the tolerance to several disinfectants that might increase the ability of AMR-carrying isolates to persist in the environment [56]. Quaternary ammonium compounds are widely used as disinfectant in farm environments. It has been observed that *qac* genes are often associated with multidrug-resistant isolates [57]. Thus, they might support the evolution (i.e., adaptation to specific environmental conditions) of bacterial resistance to multiple antimicrobial agents. Here, the presence of the biocide resistance genes reveals another risk harboured by the *qnr*-carrying isolates, as it represents an additional determinant for resistances against biocides.

### 3.4. Virulence Genes Associated with qnr-Carrying E. coli

As this study was based on commensal *E. coli*, the number of virulence-associated genes in the isolates was expected to be low. In our samples, between 34 and 108 potential virulence factors (according to the Virulence Factor Database) per isolate were identified. Ninety-two isolates were found to carry *fimH*, a D-mannose-specific adhesin, type 1 fimbriae-encoding gene. Frequent detection of the potential surface virulence factors *fimH* is quite common among *E. coli*. However, FimH mediates adherence to cells and, therewith, helps the formation of bacterial biofilms [58]. It confers the possibility of colonisation and, when certain mutations occur, can represent a virulence factor [59,60]. Additionally, other surface virulence factor encoding genes such as *afa* (*n* = 2), *focG* (*n* = 1), *paa* (*n* = 1), *pap* (*n* = 1), and *saf* (*n* = 1) were found. Moreover, we detected one isolate with *eae,* an intimin-encoding gene. The protein, encoded by *eae*, plays a critical role on the intestinal colonisation and, therefore, STEC (shiga toxin-producing *E. coli*) or another aggregative *E. coli* pathogenesis [61]. Some isolates harboured toxin genes or toxin subunits such as *astA* (*n* = 16), *cdtA* (*n* = 3), *cnf1* (*n* = 3), *eltA* (*n* = 1), and *faeC* (*n* = 1). The presence of *astA* has been detected in subgroups of enteroaggregative *E. coli* [62]. Further, two isolates possessed the *hlyA* gene, an important secretory virulence factor. Therewith, the presence of the virulence genes was unrelated to the different monitoring programs from where the *E. coli* was isolated. Thus, we showed that important virulence factors could sporadically occur in *qnr*-positive *E. coli* isolates. As many virulence factors are also located on mobile genetic elements, like plasmids, their potential spread with resistance determinants should be taken into account. Thus, through horizontal gene transfer, not only the resistance genes but additional virulence factors are spread. In these cases, antibiotic treatment failure, due to resistance, may give rise to potential impacts of certain virulence factors; therewith, representing an evolutionary pathway to pathogenicity [63]. However, most of the virulence factors detected in this study represent individual components of different complex systems. As the different virulence factors of *E. coli* are quite complex in their interaction, they may not have a high impact on the isolate’s pathogenicity on their own [64].

### 3.5. In-Silico-Based Prediction of Plasmids Types Carrying qnr

Plasmids play a major role in bacterial evolution and resistance gene transmission. Understanding factors influencing plasmid composition and evolution are essential for reliable assessments. Therefore, we detected the best-matching references to our *qnr*-carrying plasmids with the refSNPer tool. Therewith, reference plasmids that were covered by up to 100% and 90% were determined for 31 and 35 datasets, respectively, to their reference plasmids. Four WGS datasets showed no significant matches (best reference < 50% coverage) to any *qnr*-plasmid genome of the reference database and could be considered as new plasmids. The most frequently detected references were plasmid tig00003056 of *E. coli* strain AR_0162 (NZ_CP021681, *n* = 15), plasmid C of *E. coli* strain D9 (NZ_CP010155, *n* = 8), an unnamed plasmid of *Shigella flexneri* 1a strain0670 (NZ_CP020088, *n* = 8), and pKpvST101_6 of the *Klebsiella pneumoniae* strain KpvST101_OXA-48 (NZ_CP031373, *n* = 6).

Plasmids exhibiting nucleotide similarities of >80% to the best matched plasmid tig00003056 (NZ_CP021681) have been described in hospitalised patients in the USA (CP026200.1, CP044008.1), the UK (LT906492.1, LT882487.1), in Taiwan (CP046430.1), and in Pakistan (CP040574.1). Plasmid C (NZ_CP010155)-like genomes were found in China and Japan, isolated from wastewater (CP035315.1, CP045998.1, AP019678.1, MT219825.1, CP051432.1, CP046002.1) or dog faeces (NZ_CP010155). Both plasmids were mainly isolated from *E. coli* but also found in other *Enterobacteriaceae*. This geographical spread, as well as the different reservoirs, provide no further evidence on a common source/origin for *qnr*-carrying plasmids. In addition, NZ_CP020088-like plasmids seemed broadly distributed. They have been detected in Brazil (MK965545.1) and Norway (MH507589.1) in chicken and turkey meat, and in rook faeces in the Czech Republic (KF362122.2, MH121702.1). However, they also have been isolated from hospitalised patients in China (CP020088.1, KJ201886.1, CP012734.1, and CP020341.1). With NZ_CP020088-like plasmids being reported mainly in poultry origin, it supports our findings of this plasmid in the livestock reservoir. NZ_CP031373 was detected in the Netherlands (KX618696.1), Czech Republic (MH594478.1), and the UK (CP031373.2). Overall, the best matched plasmid-references to our identified *qnr*-carrying plasmids mainly originate from *E. coli* (*n* = 50) and *Shigella flexneri* (*n* = 20). However, similar plasmid-types were also identified in a broad range of other *Enterobacteriaceae* like *Enterobacter*, *Salmonella*, and *Serratia*. The broad distribution of these plasmids, closely related to our identified *qnr*-carrying plasmids, demonstrates the high ability of spread among *Enterobacteriaceae*. The diverse host adaption is clear evidence for a broad host spectrum of *qnr*-plasmids. Further, the distant locations of plasmid isolation demonstrate the putative exchange of global resistance transfer over plasmids, especially for *qnr* here.

As shown in Table 4 (and Table S4), the most abundant plasmid replicon types among our identified *qnr* reference plasmids were IncN (*n* = 12), IncY (*n* = 19), as well as a combination of IncX1 and IncX3 (*n* = 29). However, a total of 21 different plasmid-type combinations were identified. Therewith, IncN plasmids are reported as broad host range types, with the ability for conjugative transfer and the carriage of drug resistance genes. Close phylogenetic relationships from environmental and clinical samples are described for IncN plasmids [65]. Further, IncN plasmids are known for carrying a great variety of resistance genes against extended-spectrum β-lactams, sulphonamides, quinolones, aminoglycosides, tetracyclines, and streptomycin [66]. Here, we found the IncN reference plasmid to carry the *bla*_TEM-1_ resistance gene, encoding β-lactamase. IncN is widely found in *Enterobacteriaceae* and recognised as conjugative. Plasmids from the IncY type are known for frequently harbouring *bla*_CTX-M-5_ or *bla*_SHV-2_ resistance genes, associated with ESBL development [66]. Further, plasmids of these group have been shown to carry *mcr*-1 [67]. Here, we found the reference plasmid from the IncY group to harbour multiple resistance genes, including *bla*_CTX-M-5_ and *bla*_TEM-1_. Although plasmids belonging to the IncX group are known to be narrow host range plasmids, commonly found in *Enterobacteriaceae*, they often carry a wide spectrum of multidrug resistance enabling genes and were often found in the guts of animals [68]. Genes encoding carbapenemases as well as the colistin resistance genes *mcr*-1 and *mcr*-2 are frequently reported on IncX plasmids [69,70]. Dobiasova and Dolejska [71] found that IncX plasmids are widely distributed in *E. coli* in European animals and predominantly associated with (fluoro)quinolone resistance genes, particularly with *qnrS*. All of the prevalent plasmid incompatibility (Inc)-groups were often associated with multiple resistance genes besides *qnr*, which increases the risk of the dissemination of those plasmids. As shown in Table 4, the plasmids from the IncX group did carry multiple resistance genes next to *qnrS*. Thus, we detected *bla*_TEM-1_ and *bla*_SHV_ located on the same plasmids. However, while we identified certain clusters of the mentioned plasmid Inc-groups, the *qnr* gene was rather broadly distributed over different plasmid types.

**Figure 3:**
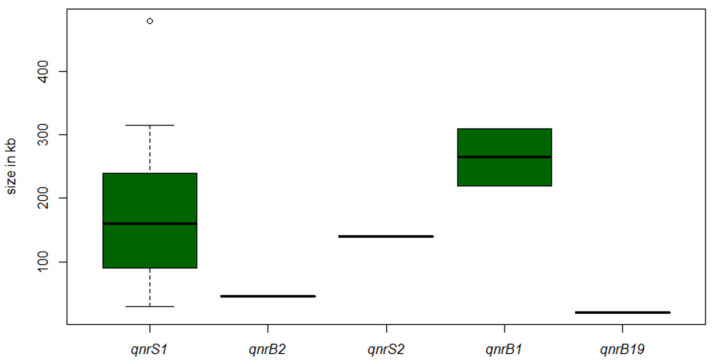
Boxplots of the size distribution of analysed plasmids harboring *qnr*, estimated through S1-PFGE. The white circle represents an outlier.

Further, we examined the size distribution of extrachromosomal DNA elements through S1-PFGE analysis and successive DNA–DNA hybridisation against the different *qnr* genes. Overall, we found a broad size diversity of plasmids carrying *qnr* (Figure 3).

The *qnrS1* gene was detected on plasmids ranging between 30 kb and 480 kb. Once again, this emphasises the ability of *qnr* to combine with different plasmid types of various sizes and, therewith, the adaptability to different niches.

Apart from *qnr*, the most frequently found resistance genes on the same reference plasmid were *aph(3″)-Ib*, *aph(6)-Id*, *bla*_CTX-M-15_, *bla*_TEM-1_, and *sul*2, independent of the monitoring program. The occurrence of this resistance gene combination was not exclusively for a certain Inc group, but rather broadly distributed. Hence, *qnr* genes are detectable on plasmids of various sizes and are seldom the only resistance gene located on the plasmid, but instead related to different resistance genes. The transfer of plasmids carrying *qnr* has been described as associated with the transfer of genes leading to multidrug resistance [72]. This suggests that provoking *qnr* resistance by overuse of an antimicrobial agent can support the expansion of multidrug-resistant isolates in livestock and food sources.

### 3.6. Frequent Detection of Point Mutations in the gyrA, parC and parE Genes of qnrS-Carrying E. coli

In our investigations, we detected isolates with phenotypes resistant against ciprofloxacin but lacking phenotype resistance against nalidixic acid (Table S2). Resistance to fluoroquinolones without resistance to quinolones is mainly associated with mutations in the chromosome within the *gyrA* and *parC* genes [58,59]. Further, the EFSA assumed that the resistance to ciprofloxacin without resistance against nalidixic acid indicates an increasing occurrence of plasmid-mediated quinolone resistance [2]. In general, besides PMQR genes, mutations in genes encoding DNA gyrase (*gyrA* and *gyrB*) and topoisomerase IV (*parC* and *parE*) are highly associated with increased (fluoro)quinolone resistance. Previous studies showed that a high level of resistance to (fluoro)quinolones is mainly associated with mutations in the *gyrA* and an additional mutation in the *parC* gene in *E. coli* [73,74]. In this study, 16 out of 103 *qnr*-carrying isolates were identified to exhibit a point mutation in the QRDR regions of *gyrA* (*n* = 9, two mutations; *n* = 5, one mutation) or *parC* (*n* = 2, two mutations; *n* = 10, one mutation) and *parE* (*n* = 4, one mutation) genes. Subsequently, these 16 isolates had high MIC values for nalidixic acid (mostly > 128 mg/L) and ciprofloxacin (mostly > 8 mg/L). Interestingly, we also detected three other isolates with a MIC for nalidixic acid > 128 mg/L, but no alteration in the chromosomal genes associated with (fluoro)quinolone resistance. However, all isolates but one had a point mutation in the *parC* region leading to the amino acid substation E62K. This strongly suggests an influence of this mutation for the (fluoro)quinolone resistance in *E. coli*. Vingopoulou et al. [75] described this ParC E62K substitution previously. They detected this new amino acid exchange in enrofloxacin-resistant *E. coli* isolated from dog otitis and faecal samples. As enrofloxacin is classified to the group of the (fluoro)quinolones, this would support the hypothesis of the E62KL exchange enhancing the (fluoro)quinolone resistance. In total, 87 of the analysed isolates were (fluoro)quinolone-resistant but carried only a *qnr* gene without any other PMQR genes associated with the development of the resistance. Possibly, the presence of *qnr* genes is sufficient to increase the MIC for NAL to a degree of resistance. One could also consider the development of yet unknown genes or point mutations in *E. coli* involved in this (fluoro)quinolone resistance. This clearly demonstrates the urgency of monitoring for PMQR to estimate resistance against (fluoro)quinolones and the possible capability of *qnr* to enhance (fluoro)quinolone resistance in *E. coli*.

## 4. Conclusions

In this study, we determined the prevalence of *qnr*-genes among (fluoro)quinolone-resistant *E. coli* from livestock and food in Germany and analysed the potential risks associated with the dissemination of the respective plasmids. While the prevalence of 3.7% of *qnr*-carrying isolates within (fluoro)quinolone-resistant *E. coli* was rather low, the risk linked with *qnr*-positive isolates is due to other reasons. Most of the *qnr*-positive *E. coli* also carried other resistance genes leading to a multidrug resistance phenotype of the isolate, especially when *E. coli* isolates from the ESBL-monitoring were analysed. Next to the occurrence of resistance genes, we detected genes leading to pesticide resistance or virulence genes within the same *qnr*-positive isolate. Further, we found that *qnr*-plasmids were widely distributed. Hence, the spread of the *qnr-*plasmid is not restricted to specific matrices or certain Inc groups. Further, we could confirm findings reporting that reported that the sole presence of *qnr* can lead to phenotypic (fluoro)quinolone resistance. We found isolates being resistant to nalidixic acid and ciprofloxacin that only carried a *qnr* gene without further PMQR or point mutations in the respective area of the chromosome. Since *qnr* is mostly identified on mobile genetic elements, this finding stresses the possible spread of this resistance determinant. The outgoing risk from *qnr* genes needs to be taken seriously, especially when evaluating (fluoro)quinolone resistance in *E. coli* isolated from livestock and food.

## Figures and Tables

**Table 1:** Occurrence and frequencies of determined *qnr* genes within *qnr*-carrying, (fluoro)quinolone-resistant *E. coli* isolates.

Gene *^1^	Gene *^2^	Occurrence	Frequency ^#^
*qnrA*	*qnrA1*	1	1.0%
*qnrB*	*qnrB1*	3	5.8%
	*qnrB2*	1	
	*qnrB19*	2	
*qnrS*	*qnrS1*	92	92.2%
	*qnrS2*	3	
*qnrVC*	*qnrVC4*	1	1.0%

*^1^: determined with PCR, *^2^: determined with WGS, ^#^ frequency of genes as determined by PCR.

**Table 2:** Absolute number of the phenotypic resistance of *qnr*-carrying isolates and absolute number of isolates gained from the respective matrix. In brackets the absolute number of isolates from the ZoMo-/ESBL-Monitoring is indicated.

Matrix	Matrix Occurrence	AMP	AZI	CHL	CIP	COL	FOT	GEN	MERO	NAL	SMX	TAZ	TET	TMP
**faeces, veal calves**	56 (52/4)	54 (51/3)	4 (4/0)	19 (17/2)	55 (52/3)	1 (1/0)	53 (52/1)	6 (6/0)	1 (1/0)	8 (7/1)	37 (36/1)	52 (52/0)	48 (44/4)	40 (37/3)
**faeces, deer**	1 (1/0)	1	0	0	1	0	1	0	0	0	1	1	1	1
**faeces, pigs**	38 (24/14)	38 (24/14)	6 (5/1)	11 (9/2)	37 (24/13)	0	27 (24/3)	4 (4/0)	0	10 (7/3)	22 (18/4)	27 (24/3)	22 (17/5)	20 (17/3)
**meat, veal**	2 (2/0)	2	0	2	2	0	2	0	0	1	1	1	1	2
**meat, deer**	1 (1/0)	1	1	0	1	0	1	1	0	0	1	1	1	1
**meat, pork**	2 (0/2)	1	0	2	2	0	0	0	0	0	1	0	1	2
**minced meat**	3 (0/3)	3	0	0	3	0	1	0	0	0	0	1	2	0

AMP—Ampicillin, AZI—Azithromycin, CHL—Chloramphenicol, CIP—Ciprofloxacin, COL—Colistin, FOT—Cefotaxime, GEN—Gentamicin, MERO—Meropenem, NAL—Nalidixic acid, SMX—Sulfamethoxazole, TAZ—Ceftazidime, TET—Tetracycline, TMP—Trimethoprim.

**Table 3:** *p*-value for co-occurrence of selected resistance genes and *qnr* genes in ESBL-monitoring isolates; *p*-values below 0.05 are highlighted in red and represent statistical significance. The value 1 reflects that the two genes were not detected.

	*qnrA1*	*qnrB1*	*qnrB2*	*qnrB19*	*qnrS1*	*qnrS2*
*aadA1*	0.1625	0.00348101	1	1	0.00462503	0.41693038
*aph(3′)-lia*	1	1	1	1	0.1125	0.0375
*aph(3′)-XV*	0.0125	1	1	1	0.1125	1
*arr-3*	1	1	1	1	0.011392405	0.00094937
*bla*_ACC-1_	0.0125	1	1	1	0.1125	1
*bla*_CTX-M-65_	1	1	1	1	0.060414269	0.00559883
*bla*_OXA-1_	1	0.00012171	1	1	0.000000524	0.00925024
*bla*_VIM-1_	0.0125	1	1	1	0.1125	1
*catA1*	0.0625	0.00012171	1	1	0.000377371	1
*catB2*	0.0125	1	1	1	0.1125	1
*catB3*	1	1	1	0.0375	0.001022395	0.00282376
*dfrA25*	1	1	0.0125	1	0.1125	1
*floR*	0.25	1	1	1	0.038946034	0.01387537
*mef(C)*	1	0.10966407	1	1	0.032132425	0.10966407
*mph(A)*	0.2125	1	1	1	0.090321713	0.00827653
*mph(G)*	1	0.10966407	1	1	0.032132425	0.10966407
*qacE*Δ1	0.2	0.49289192	0.2	0.2	0.001541798	0.10029211
*sul1*	0.175	0.44303798	0.175	0.175	0.006847169	0.44303798

**Table 4:** Inc group and resistance genes of the best matching reference plasmid to the most prevalent plasmids carrying *qnr*-genes detected in this study. Identified with RefSNPer.

Plasmid Type and Resistance Genes on Matching Reference	Frequency
IncN	**∑** 12
*aac(3)-IId, qnrS1*	1
*bla*_TEM-1_ *, qnrS1*	5
*qnrB19*	1
*qnrS1*	5
IncR, IncX1	**∑** 1
*aadA2, blaTEM-1, dfrA12, floR, qnrS1, sul2, tet(A), tet(M)*	1
IncX1	**∑** 9
*aph(3′)-Ia, floR, qnrS2*	8
*bla*_TEM-1_ *, qnrS1, tet(M)*	1
IncX1, IncX3	**∑** 14
*bla*_TEM-1_ *, qnrS1*	14
IncX3	**∑** 6
*bla*_SHV_ *, qnrS1*	6
IncY	**∑** 19
*aph(3″)-Ib, aph(6)-Id, bla*_CTX-M-15_ *, bla*_TEM-1_ *, qnrS1, sul2, tet(A)*	15
*aph(3″)-Ib, aph(6)-Id, bla*_CTX-M-15_ *, bla*_TEM-1_ *, dfrA14, qnrS1, sul2, tet(A)*	2
*aph(3″)-Ib, aph(6)-Id, bla*_CTX-M-15_ *, bla*_TEM-1_ *, qnrS1, sul2*	2

## Data Availability

Data is contained within the article or supplementary material.

## Supplementary Materials

The following are available online at https://www.mdpi.com/article/10.3390/microorganisms9061308/s1. Supplemental Figure S1: Grouped bar chart of total number of resistant isolates per antimicrobial agent and matrix. Table S1: Sequences, annealing temperatures, and references of used primers for *qnr* detection. Table S2: Matrix and MIC values of investigated (fluoro)quinolone-resistant and *qnr*-positive *E. coli* isolates, as well as their resistance profile. Table S3: *p*-value of respective Fisher’s exact test; *p*-values < 0.05 represent statistical significance and are highlighted in red. The value 1 reflects that the two genes were not detected; columns with only 1 as value are not presented. Table (A) shows the correlation for genes detected in the ESBL-monitoring isolates. Table (B) shows the correlation for genes detected in the ZoMo isolates. Table S4: Inc-group and resistance genes of all the best-matching reference plasmids to the plasmids carrying *qnr* genes detected in this study. Identified with RefSNPer.
